# Prognostic Value of the Combination of HB (hemoglobin) and CEA in Resectable Gastric Cancer

**DOI:** 10.7150/jca.67600

**Published:** 2022-04-11

**Authors:** Xinyue Qiu, Cheng Shen, Wenjing Zhao, Xunlei Zhang, Dakun Zhao, Yueyue Zhu, Guoxing Li, Lei Yang

**Affiliations:** 1 Cancer Research Center, Affiliated Tumor Hospital of Nantong University, Nantong Jiangsu, China; 2 Department of Computer Science and Engineering, Tandon School of Engineering, New York University, Brooklyn, NY 11201, US; 3 Department of Oncology, Affiliated Tumor Hospital of Nantong University, Nantong Jiangsu, China; 4 Department of Oncology, Affiliated Hospital of Nantong University, Nantong Jiangsu, China; 5 Department of Surgery, Affiliated Tumor Hospital of Nantong University, Nantong Jiangsu, China

**Keywords:** hemoglobin, CEA, gastric carcinoma, patients, survival

## Abstract

**Objective:** In order to investigate the prognostic value of a novel biomarker combining serum carcinoembryonic antigen (CEA) and hemoglobin (HB) levels in patients with resectable gastric cancer.

**Introduction:** This retrospective study assessed the relationship between CEA, hemoglobin levels, a novel combined prognostic biomarker (HB-CEA) and clinicopathological features of gastric cancer. Their prognostic values in gastric cancer were also analyzed.

**Materials and Methods:** This retrospective study evaluated the CEA, hemoglobin levels and clinicopathological features of patients with resectable gastric cancer. Kaplan-Meier curves, univariate and multivariate Cox proportional models were used to determine the prognostic significance of these factors for overall survival (OS) in the training and validation sets (n=353 and n=388, respectively). Based on optimal cutoff values of CEA and hemoglobin (3.395 ng/mL and 125.5 g/L, respectively), patients were stratified into three groups: HB-CEA=0, 1, and 2 (CEA <3.395 ng/mL and HB ≥125.5 g/L; CEA ≥3.395 ng/mL or HB <125.5 g/L; and CEA ≥3.395 ng/mL and HB <125.5 g/L, respectively).

**Results:** The area under the curve was larger for HB-CEA than for either HB or CEA alone (training set: 0.677, 0.650, and 0.629; validation set: 0.670, 0.605, and 0.605, respectively). HB-CEA was strongly associated with age, tumor size, differentiation, pathological TNM stage (pTNM), depth of tumor invasion, lymph node metastasis, and survival status (all *p*<0.05). A higher HB-CEA score correlated with poor survival (Kaplan-Meier curves, all *p*<0.05). Multivariate analysis showed that HB-CEA was an independent prognostic factor for OS (*p*<0.05).

**Conclusion:** Preoperative HB-CEA, as a potential novel hematological biomarker, can predict the progression of gastric cancer and the prognosis of patients, and is of great value in guiding clinical practice. Therefore, patients with a higher HB-CEA score should receive more extensive follow-up for early detection and intervention of tumor progression.

## Introduction

Worldwide, gastric cancer ranks fifth among all cancers and is the third most common cause of cancer mortality 1, but ranks second among all cancers and is the third most common cause of cancer mortality in the People's Republic of China 2. As early-stage gastric cancer is often asymptomatic and difficult to detect, most patients have advanced-stage gastric cancer at the initial diagnosis. Radical gastrectomy is the mainstay of treatment for early-stage gastric cancer. However, different risk groups and additional potential prognostic biomarkers need to be identified for patients with gastric cancer who have different physical conditions and disease states. Therefore, the identification of accurate prognostic factors and the development of validated scoring systems to predict survival outcomes in patients with gastric cancer are essential for developing individualized treatment plans for patients with gastric cancer.

Increasingly, several studies have shown that hematological biomarkers, determined via rapid and easy testing, can predict the prognosis of patients with gastric cancer. The commonest hematological biomarkers for gastric cancer include hemoglobin (HB), neutrophil-to-lymphocyte ratio (NLR) 3, platelet-to-lymphocyte ratio (PLR) 3, and tumor markers 4. Anemia is a common symptom in patients with cancer, of which the most intuitive reflection is a decrease in the hemoglobin level that often has a multifactorial causation 5. Cancer-related anemia occurs frequently in malignancies and is one of the commonest comorbidities. The prevalence of pretreatment anemia is high and ranges from 30% to 90% in various cancers 6. The causes of cancer-related anemia include tumor-related blood loss, bone marrow involvement, cytokine-mediated disorders, and nutritional deficiencies of iron or folic acid 7. The widespread pretreatment anemia adversely affects the treatment and prognosis of patients with cancer 8, 9. In the past decade, evidence has accumulated to indicate that anemia predicts poor patient survival in many cancers, including gastric cancer 10,11.

Tumor markers can be used as indicators for determining the diagnosis, treatment, and prognosis of patients with gastric cancer. Serum levels of the tumor markers (e.g. carcinoembryonic antigen [CEA], carbohydrate antigen CA199 and CA 72-4, and alpha fetoprotein [AFP]) are elevated in some patients with gastric cancer 12. CEA is a common serum marker for malignant gastrointestinal tract tumors and facilitates a diagnosis of gastric cancer. Moreover, the expression of CEA is an independent risk factor for poor prognosis in gastric cancer 13. Elevated preoperative CEA has been proposed as a predictor of overall survival (OS) in early-stage gastric cancer 13. The combination of CEA with CA19-9 and CA72-4 has been reported to predict survival in gastric cancer and prove important for subsequent treatment-related decisions 14. Accordingly, we proposed the hypothesis that a combination of tumor markers and individual patient differences can be analyzed together to predict the survival of patients with gastric cancer.

Therefore, the aim of this study was to assess the clinical value of the novel combination prognostic biomarker (HB-CEA) for gastric cancer. In addition, we assessed the relationship between HB-CEA and the clinicopathological features of gastric cancer.

## Materials and Methods

This study involved a retrospective evaluation of patients with primary gastric cancer who underwent radical gastrectomy between 2007 and 2016 (training set) and between 2010 and 2012 (validation set) at the Affiliated Tumor Hospital of Nantong University and the Affiliated Hospital of Nantong University. The inclusion criteria: (1) availability of complete patient clinical data (including sex, age, tumor location, differentiation, diameter of lesion, cancer embolism, nerve invasion, pathological TNM stage (pTNM), depth of invasion, lymph node metastasis, and survival status); (2) without coexistence of other tumors and hematological diseases; (3) received radical gastrectomy. The exclusion criteria: (1) received neoadjuvant chemotherapy or radiotherapy before surgery; (2) with metastases to lung, liver and other organs in preoperative imaging examinations, and distant metastasis in intraoperative examinations; (3) hepatic or renal insufficiency; (4) acceptance of palliative surgery. Thus, 741 patients were enrolled in the present study.

This retrospective study was approved by the Medical Institutional Ethics Committee of the Affiliated Tumor Hospital of Nantong University and the Affiliated Hospital of Nantong University, Jiangsu province. The data were anonymous and, therefore, the requirement for informed consent was waived.

### Data Collection

Details of the clinicopathological information of the participants, including sex, age, tumor location, differentiation, diameter of lesion, cancer embolism, nerve invasion, pathological TNM stage (pTNM), depth of invasion, lymph node metastasis, and survival status, were obtained from the medical records. OS was defined as the interval from the date of the surgery to the date of death or the last follow-up. Pretreatment fasting peripheral blood samples were collected within 1 week preceding the surgery and were processed within 48 hours after collection to assess HB-CEA levels, liver and kidney function, coagulation function, and other predefined indicators. Clinical data of the patients with gastric cancer were collected from the hospital information system. Staging was performed according to the TNM classification of the American Joint Committee on Cancer (8^th^ Edition).

### Statistical Analysis

The optimal cutoff values for HB, CEA, and other variables were obtained via the receiver operating characteristic (ROC) curve analysis, and the participants were then divided into two study groups according to the cutoff. Additionally, the participants were divided into three groups according to the HB-CEA score: HB-CEA=0 (CEA <3.395 ng/mL and HB ≥125.5 g/L), HB-CEA =1 (CEA ≥3.395 ng/mL or HB <125.5 g/L), and HB-CEA=2 (CEA ≥3.395 ng/mL and HB <125.5 g/L) (Table 1). The chi-square or Fisher's exact test was used to analyze categorical variables. In the training set, propensity score matching (PSM) was performed to reduce selection bias and to quantify the possible association between HB-CEA and OS. The Kaplan-Meier method was used for survival analysis. Variables found to be significant in univariate analysis were included in multivariate analysis. Multivariate Cox model was used to analyze the factors that independently affect the survival and prognosis of gastric cancer patients. All statistical analyses were performed using SPSS 26.0 and R Package (http://www.r-project.org/). *P*<0.05 was considered indicative of statistical significance.

## Results

### Patient Characteristics

The training set comprised 353 patients with gastric cancer, including 117 (33.1%) women and 236 (66.9%) men; 179 (50.7%) and 174 (49.3%) participants were older than 65 years and aged 65 years or younger, respectively. The validation set comprised 388 participants with gastric cancer, including 122 (31.4%) women and 266 (68.6%) men; 165 (42.5%) and 223 (57.5%) participants were older than 65 years and aged 65 years or younger, respectively. The clinicopathological characteristics of the participants in the study cohorts are summarized in Table 2.

### Optimal Cutoff Values of CEA and HB

**Figure 1:**
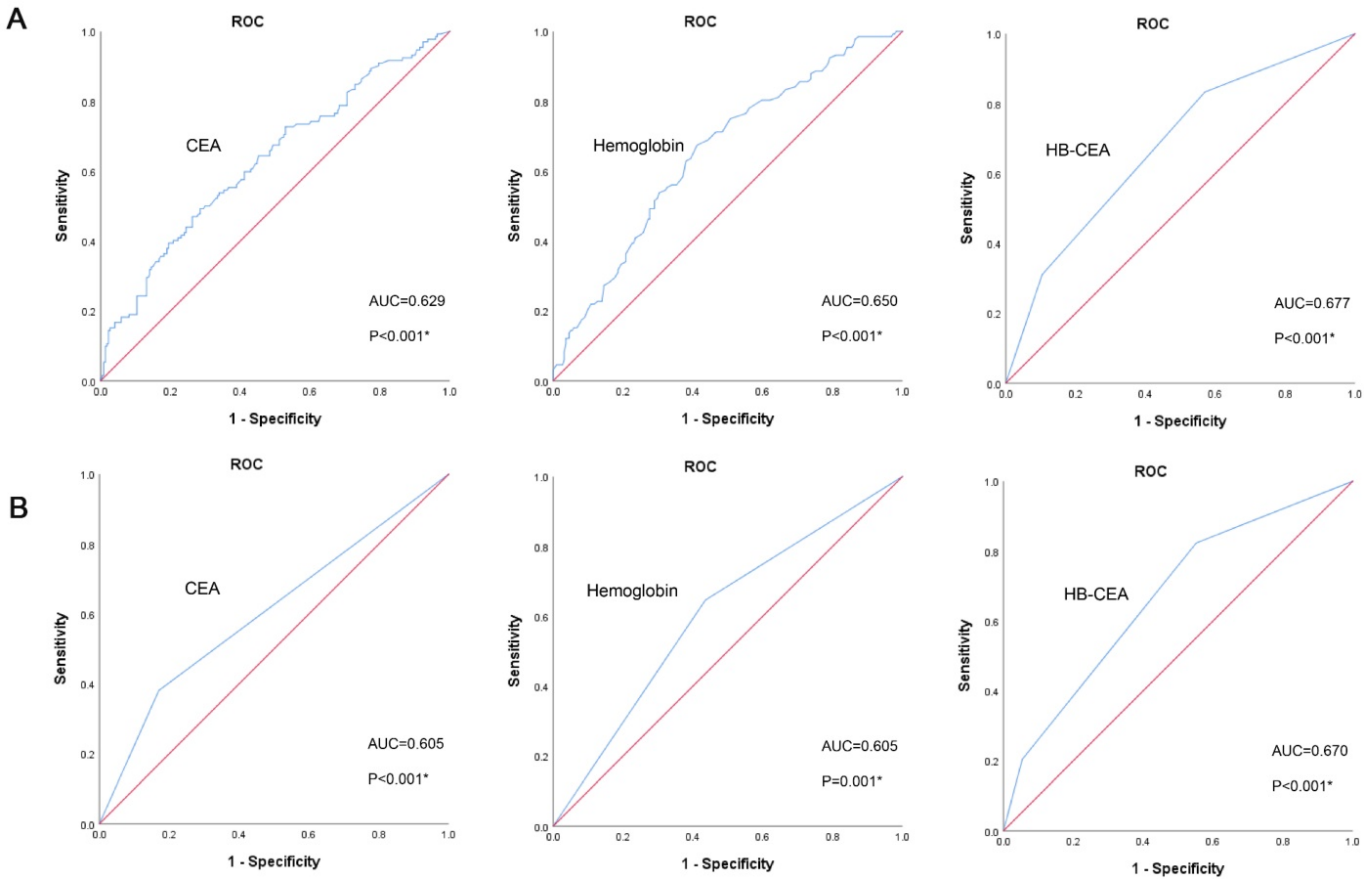
Receiver operating characteristics curve analysis of HB, CEA and HB-CEA for OS in GC patients (the training set, **A**; the validation set, **B**). Note: HB-CEA: HB≥125.5 and CEA<3.395 represent 0; CEA≥3.395 or HB<125.5 represent 1; CEA≥3.395 and HB<125.5 represent 2. Abbreviations: GC: gastric cancer; CEA: carcinoembryonic antigen; HB: hemoglobin; OS: overall survival.

In the training set, the optimal cutoff value of CEA for OS was 3.395 ng/mL (area under curve [AUC]=0.629, 95% confidence interval [CI]: 0.569-0.690, *p*<0.001; Figure 1A). The optimal cutoff value of hemoglobin for OS was 125.5 g/L (AUC=0.650, 95% CI: 0.592-0.709, *p*<0.001; Figure 1A). Based on these cutoff values, patients were divided into two groups: high CEA (≥3.395 ng/ mL, n=120) and low CEA (<3.395 ng/mL, n=233); high HB (≥125.5 g/L, n=173) and low HB (<125.5 g/L, n=180). The AUC of HB-CEA for OS was 0.677 (95% CI: 0.619-0.734,* p*<0.001; Figure 1A), which is slightly higher than that of hemoglobin (AUC=0.650, 95% CI: 0.592-0.709, *p*<0.001; Figure 1A) and CEA (AUC=0.629, 95% CI: 0.569-0.690, *p*<0.001; Figure 1A). These results demonstrate that HB-CEA may be superior to HB or CEA alone as a prognostic marker of gastric cancer. Similar results were obtained in the validation set. The AUC of HB-CEA for OS was 0.670 (95% CI: 0.615-0.725, *p*<0.001; Figure 1B), which is slightly higher than that of hemoglobin (AUC=0.605, 95% CI: 0.548-0.663, *p*=0.001; Figure 1B) and CEA (AUC=0.605, 95% CI: 0.546-0.665, *p*<0.001; Figure 1B).

### Relationship of Preoperative HB and CEA with Clinicopathological Features

The preoperative HB and CEA were associated with various clinicopathological features of patients with gastric cancer (Table 2). In the training set, high CEA significantly correlated with sex (*p*=0.010), pathological TNM stage (pTNM) (*p*=0.006), depth of invasion (*p*=0.002), lymph node metastasis (*p*=0.011), and survival status (*p*<0.001) but not with age, tumor location, diameter of lesion, cancer embolism, nerve invasion, and differentiation (all *p*>0.05). Similarly, preoperative hemoglobin levels significantly correlated with sex (*p*<0.001), differentiation (*p*=0.041), diameter of lesion (*p*=0.003), cancer embolism (*p*=0.002), pathological TNM stage (pTNM) (*p*=0.001), depth of invasion (*p*<0.001), lymph node metastasis (*p*=0.004), and survival status (*p*<0.001), but not with age, tumor location, and nerve invasion (all *p*>0.05). However, in the validation set, high CEA significantly correlated with sex (*p*=0.004), age (*p*<0.001), diameter of lesion (*p*=0.025), pathological TNM stage (pTNM) (*p*=0.003), depth of invasion (*p*=0.006), lymph node metastasis (*p*=0.002), and survival status (*p*<0.001) but not with the tumor location, differentiation, cancer embolism, and nerve invasion (all *p*>0.05). Similarly, preoperative hemoglobin levels significantly correlated with sex (*p*<0.001), age (*p*=0.002), differentiation (*p*<0.001), diameter of lesion (*p*<0.001), pathological TNM stage (pTNM) (*p*=0.015), depth of invasion (*p*<0.001), lymph node metastasis (*p*=0.001), and survival status (*p*<0.001), but not with tumor location, cancer embolism, and nerve invasion (all *p*>0.05).

### Prognostic Significance of Preoperative HB and CEA Levels

**Figure 2:**
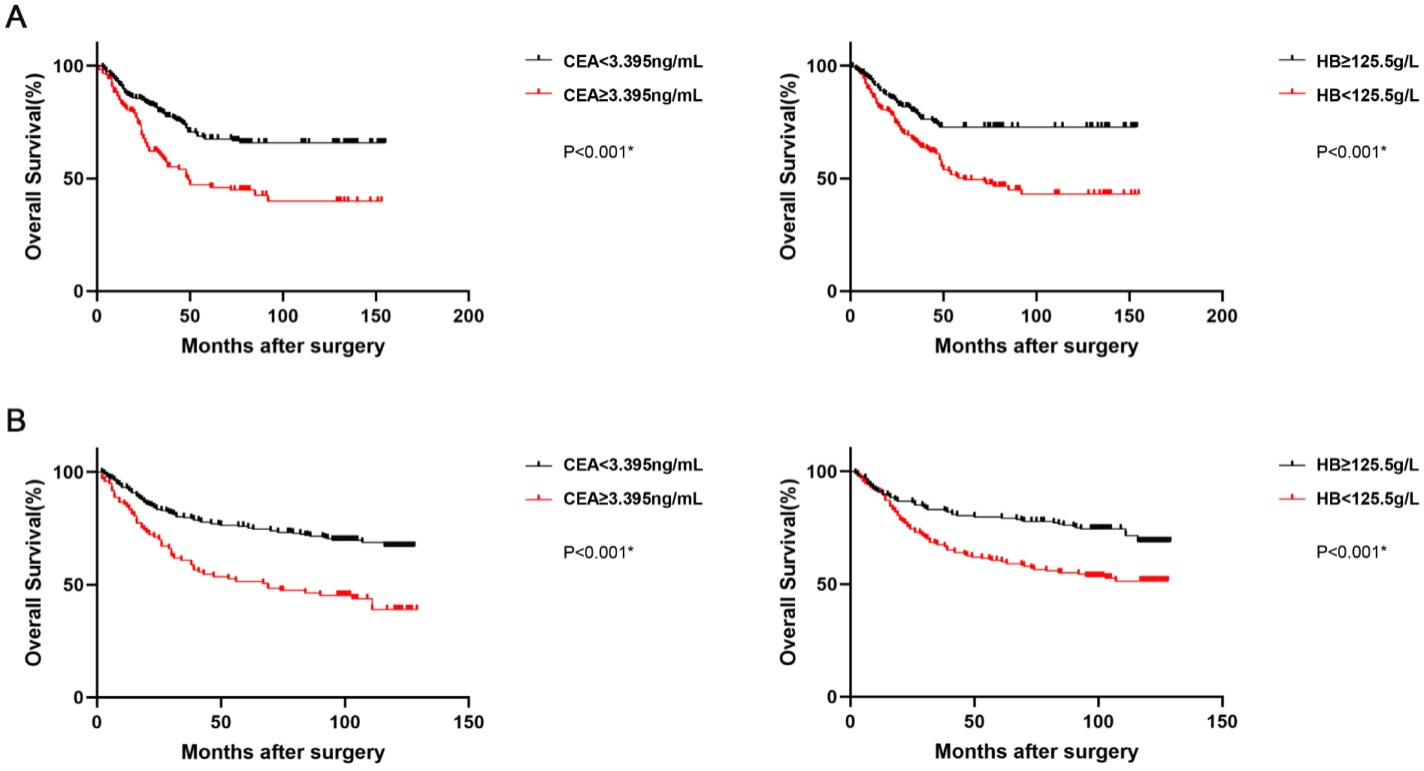
Kaplan-Meier survival curves for OS according to CEA and HB in GC patients (the training set, **A**; the validation set, **B**). Abbreviations: OS: overall survival; GC: gastric cancer; CEA: carcinoembryonic antigen; HB: hemoglobin.

The prognostic value of preoperative CEA and hemoglobin levels was demonstrated by Kaplan-Meier analysis and Log-rank test, and the overall survival rate and median overall survival time in the low CEA group were obviously higher than those in the high CEA group (*p*<0.001). Furthermore, the OS rate and median OS time in the high hemoglobin group were also obviously higher than those in the low hemoglobin group (*p*<0.001; Figure 2A). The correlation of CEA (*p*<0.001) and hemoglobin (*p*<0.001; Figure 2B) with the OS were similarly indicated in the validation set.

### Relationship of the HB-CEA Score with Clinicopathological Characteristics

We found significant intergroup differences among the three groups with regard to age (*p*=0.026), differentiation (*p*=0.014), diameter of lesion (*p*=0.005), cancer embolism (*p*=0.037), depth of invasion (*p*<0.001), lymph node metastasis (*p*=0.001), pathological TNM stage (pTNM) (*p*<0.001), and survival status (*p*<0.001) upon analyzing the relationship between HB-CEA and clinicopathological features in the training set (Table 3). Analysis of heterogeneity after PSM between the sub-cohorts stratified by the HB-CEA score cutoffs showed almost balanced distribution of the patient characteristics, including sex, age, tumor location, differentiation, diameter of lesion, cancer embolism, nerve invasion, pathological TNM stage (pTNM), depth of invasion, and lymph node metastasis, among the 64, 128, and 64 patients (HB-CEA=0, 1, and 2, respectively; *p*>0.05). Thus, the PSM-adjusted analysis could facilitate the investigation of whether the HB-CEA is associated with increased OS in a total of 256 patients. However, in the validation set, the HB-CEA score was significantly correlated with sex (*p*=0.037), age (*p*<0.001), differentiation (*p*<0.001), diameter of lesion (*p*<0.001), pathological TNM stage (pTNM) (*p*<0.001), depth of invasion (*p*<0.001), lymph node metastasis (*p*<0.001), and survival status (*p*<0.001) (Table 3).

### Univariate and Multivariate Survival Analyses

**Figure 3:**
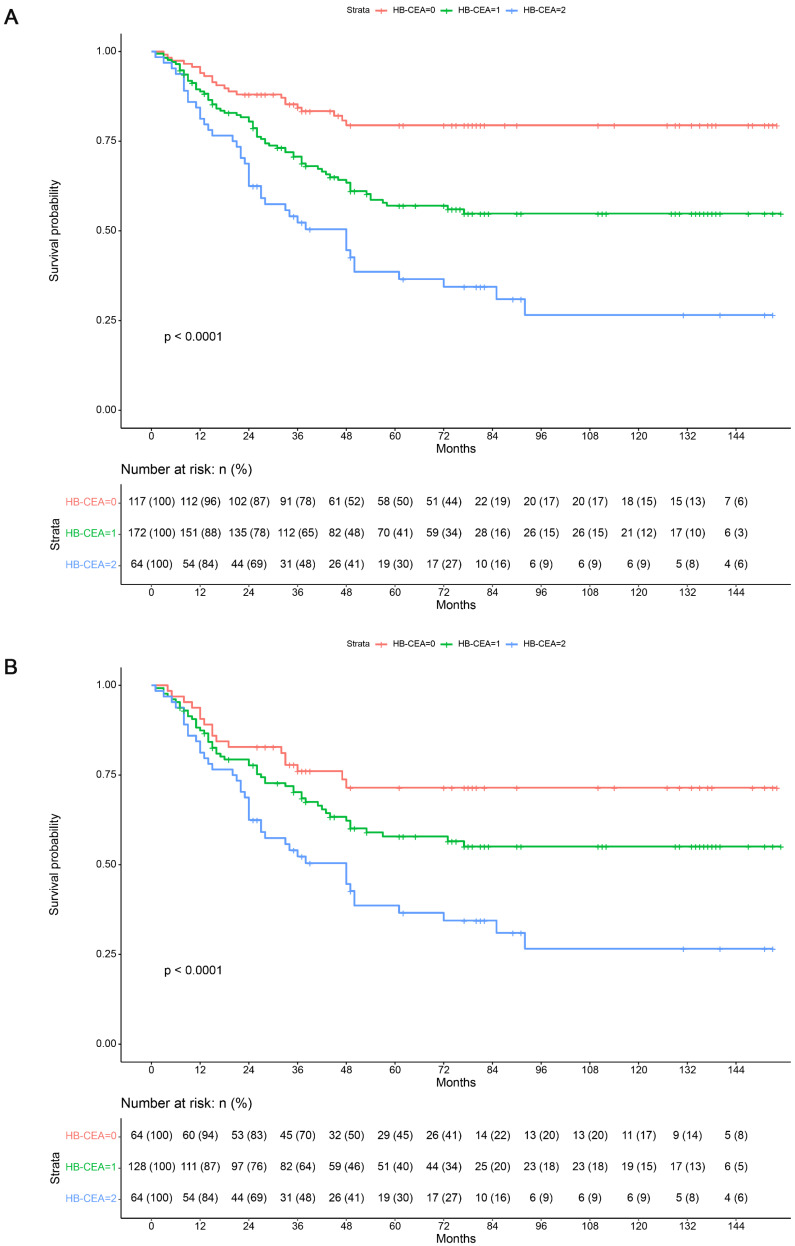
Kaplan-Maier survival curves for overall survival of patients in the training set before (**A**) and after (**B**) propensity score matching. Note: HB-CEA: HB≥125.5 and CEA<3.395 represent 0; CEA≥3.395 or HB<125.5 represent 1; CEA≥3.395 and HB<125.5 represent 2.

In order to evaluate the predictors of OS (overall survival) after radical gastrectomy, the clinicopathological characteristics were evaluated using univariate and multivariate analyses. The Kaplan-Meier analysis showed that a higher HB-CEA score was associated with worse OS (*p*<0.001; Figure 3). Before PSM, the univariate analysis revealed that differentiation (HR: 0.045, 95% CI: 0.002-0.916, *p*=0.044), diameter of lesion (HR: 1.707, 95% CI: 1.080-2.698, *p*=0.022), cancer embolism (HR: 2.069, 95% CI: 1.468-2.917, *p*<0.001), nerve invasion (HR: 1.607, 95% CI: 1.138-2.268, *p*=0.007), pathological TNM stage (pTNM) (HR: 3.243, 95% CI: 2.193-4.797, *p*<0.001), depth of invasion (HR: 2.530, 95% CI: 1.636-3.912, *p*<0.001), lymph node metastasis (HR: 3.806, 95% CI: 2.385-6.075, *p*<0.001), Hb levels (HR: 2.131, 95% CI: 1.481-3.067, *p*<0.001), CEA levels (HR: 2.061, 95% CI: 1.463-2.903, *p*<0.001), and HB-CEA (HB-CEA=1, HR: 2.362, 95% CI: 1.462-3.817, *p*<0.001; HB-CEA=2, HR: 4.339, 95% CI: 2.583-7.289, *p*<0.001) were significantly correlated with patient prognosis in the training set. Univariate analysis after PSM revealed that cancer embolism (HR: 1.863, 95% CI: 1.279-2.713, *p*=0.001), nerve invasion (HR: 1.490, 95% CI: 1.021-2.173, *p*=0.038), pathological TNM stage (pTNM) (HR: 3.219, 95% CI: 1.938-5.348, *p*<0.001), depth of invasion (HR: 1.978, 95% CI: 1.032-3.792, *p*=0.040), lymph node metastasis (HR: 4.829, 95% CI: 2.437-9.569, *p*<0.001), Hb levels (HR: 1.691, 95% CI: 1.124-2.545, *p*=0.012), CEA levels (HR: 1.814, 95% CI: 1.245-2.643, *p*=0.002), and HB-CEA (HB-CEA=1, HR: 1.615, 95% CI: 0.933-2.797, *p*=0.087; HB-CEA=2, HR: 2.915, 95% CI: 1.655-5.134, *p*<0.001) were related to the prognosis of patients (Table 4). In the validation set, univariate analysis showed age (HR: 1.430, 95% CI: 1.035-1.976, *p*=0.030), differentiation(HR: 0.358, 95% CI: 0.176-0.731, *p*=0.005),diameter of lesion (HR: 2.264, 95% CI: 1.491-3.437, *p*<0.001), cancer embolism (HR: 2.299, 95% CI: 1.492-3.542, *p*<0.001), nerve invasion (HR: 2.241, 95% CI: 1.511-3.323, *p*<0.001), pathological TNM stage (pTNM) (HR: 3.537, 95% CI: 2.511-4.982, *p*<0.001), depth of invasion (HR: 2.766, 95% CI: 1.905-4.018, *p*<0.001), lymph node metastasis (HR: 3.841, 95% CI: 2.591-5.694, *p*<0.001), Hb levels (HR: 1.968, 95% CI: 1.402-2.761, *p*<0.001), CEA levels (HR: 2.273, 95% CI: 1.628-3.173, *p*<0.001), and HB-CEA (HB-CEA=1, HR: 2.585, 95% CI: 1.671-3.999, *p*<0.001; HB-CEA=2, HR: 5.427, 95% CI: 3.203-9.195, *p*<0.001) were associated with survival (Table 4).

In the training set, the multivariate analysis before PSM showed that the HB-CEA (HB-CEA=1, HR: 1.840, 95% CI: 1.134-2.985, *p*=0.013; HB-CEA=2, HR: 3.255, 95% CI: 1.927-5.500, *p*<0.001), pathological TNM stage (pTNM) (HR: 1.800, 95% CI: 1.125-2.881, *p*=0.014), and lymph node metastasis (HR: 2.134, 95% CI: 1.217-3.741, *p*=0.008) were predictors of the clinical outcome (Table 4). Similar results were obtained with multivariate analysis after PSM. In the validation set, HB-CEA (HB-CEA=1, HR: 2.049, 95% CI: 1.313-3.198, *p*=0.002; HB-CEA=2, HR: 4.007, 95% CI: 2.335-6.877, *p*<0.001), nerve invasion (HR: 1.525, 95% CI: 1.013-2.295, *p*=0.043), pathological TNM stage (pTNM) (HR: 1.894, 95% CI: 1.225-2.928, *p*=0.004), and lymph node metastasis (HR: 1.949, 95% CI: 1.175-3.233, *p*=0.010) were associated with the prognosis of patients (Table 4).

### Nomogram and Calibration Curve for Predicting the OS of Patients with Gastric Cancer

**Figure 4:**
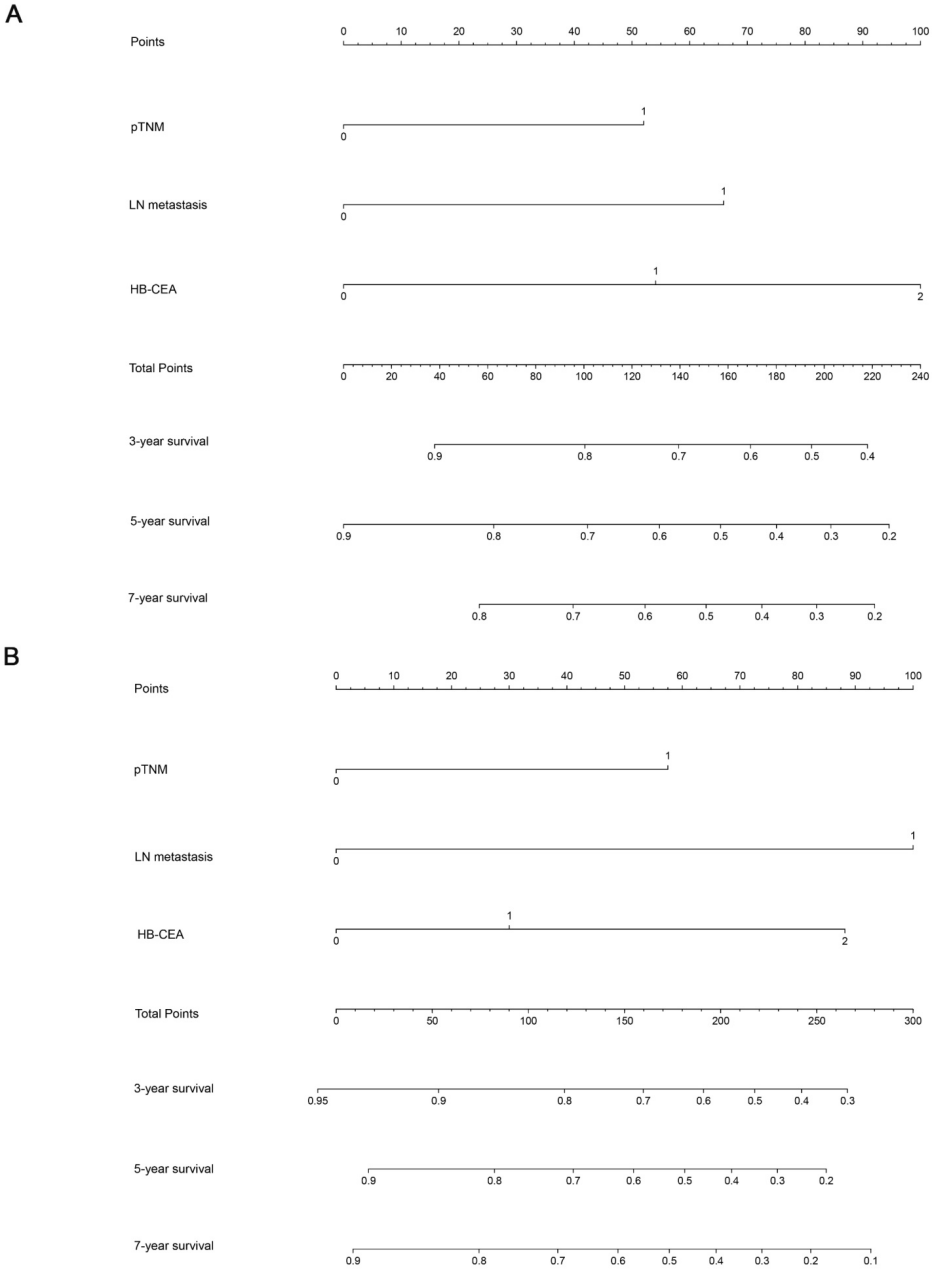
Nomograms for predicting 3-, 5-, and 7-year OS for patients in the training set (before PSM, **A**; after PSM, **B**) with the indicated prognosis factors. Summing up points from all predictors could obtain total points. The predicted probabilities of OS can be obtained by projecting the location of the total points to the bottom scales. Note: HB-CEA: HB≥125.5 and CEA<3.395 represent 0; CEA≥3.395 or HB<125.5 represent 1; CEA≥3.395 and HB<125.5 represent 2; pTNM (I-II) represent 0; pTNM (III-IV) represent 1; LN metastasis(N0) represent 0; LN metastasis(N1/N2/N3) represent 1. Abbreviations: CEA: carcinoembryonic antigen; HB: hemoglobin; pTNM: pathological TNM stage; LN metastasis: lymph node metastasis.

**Figure 5:**
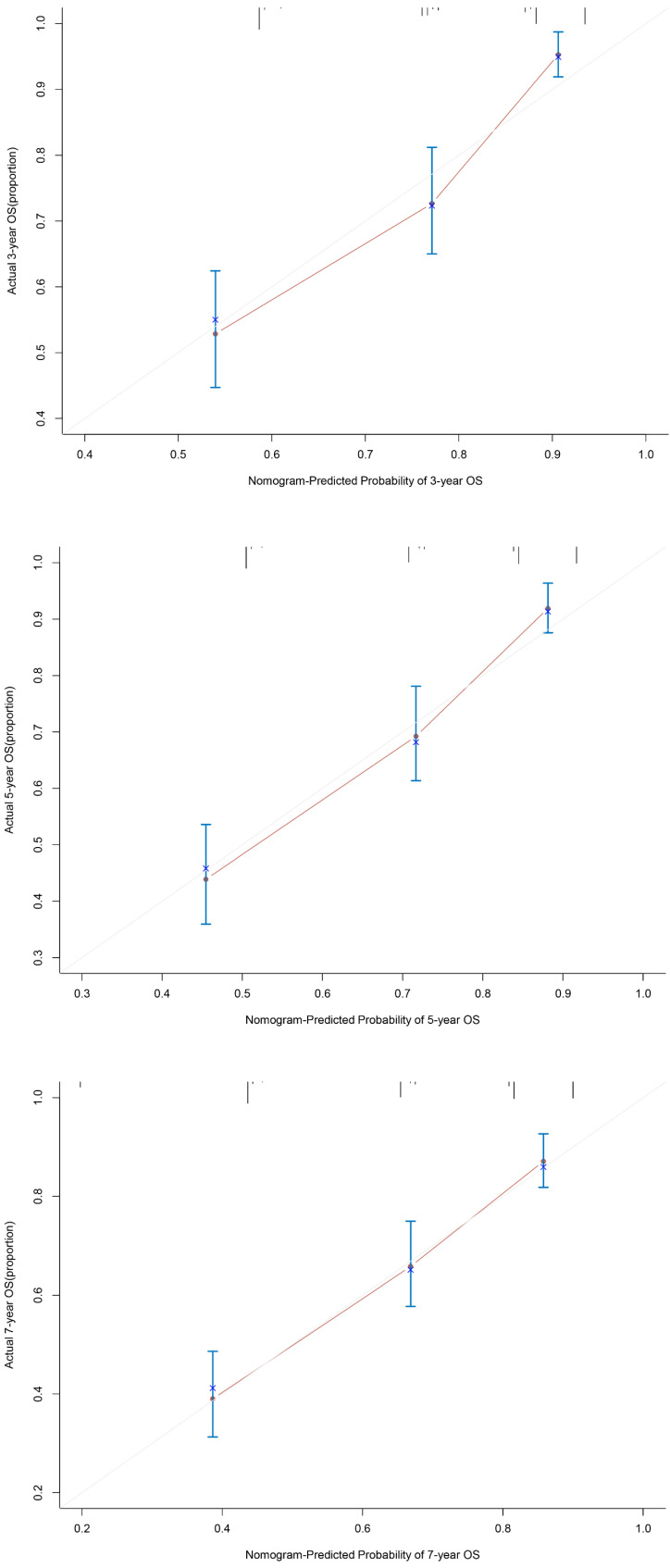
The calibration curves for predicting 3 years, 5 years and 7 years OS for GC patients in the validation set. The OS predicted by the nomogram model is plotted on the x-axis, and the actual OS is plotted on the y-axis. Abbreviations:OS: overall survival; GC: gastric cancer.

Using the abovementioned covariates identified by the statistical analyses, nomograms before and after PSM were constructed for predicting the 3-, 5-, and 7-year OS according to the training set. The score details of each nomogram predictor are shown in Figure 4. Summing the scores of all selected variables can facilitate the accurate determination of the survival probability of individual patients. The analysis of the data from the validation set showed that C-index of OS can be accurately predicted with a value of 0.723. The calibration curve showed good concordance between the predicted and observed values of 3-, 5-, and 7-year OS in the validation set (Figure 5).

## Discussion

Hematological biomarkers, such as CEA and hemoglobin, determined from peripheral blood samples are useful prognostic indicators of clinical outcomes in patients with cancer. In this study, we established the presence of a relationship between peripheral blood biomarkers and clinical outcomes in patients with resectable gastric cancer. A lower hemoglobin level or high CEA were associated with sex, larger tumor size, more advanced pathological TNM stage, deeper depth of tumor invasion, more severe lymphatic metastasis, and worse survival status. Notably, this study reveals for the first time that HB-CEA is a more effective prognostic marker than either HB or CEA alone in patients with gastric cancer scheduled to undergo radical gastrectomy. Patients with gastric cancer and an HB-CEA score of 2 had worse prognoses than those with an HB-CEA score of 0, indicating that a higher HB-CEA score is related to the tumor burden and tumor progression. Thus, HB-CEA is a promising prognostic marker in patients with gastric cancer as tumor progression is closely related to both patient and tumor characteristics.

Gastric cancer is a wasting disease, and most patients are diagnosed at an advanced stage 15. Most patients with gastric cancer suffer from malnutrition, which can exacerbate perioperative stress and further reduce the body's immunity, leading to a poor prognosis 16. Hemoglobin can reflect the nutritional status of the patient as well as the clinical condition. Therefore, it has been proposed that early enteral nutrition support can significantly enhance the cellular immune function of patients undergoing radical gastric cancer surgery, improve their nutritional status, and reduce the risk of postoperative complications 17. The current primary treatment for severe anemia is blood transfusion, which is believed to reduce the incidence of anemia-associated postoperative complications. However, transfusions are associated with a number of adverse effects, including pulmonary complications, graft-versus-host disease, and transmission of infectious diseases 18-23. Tumor markers comprise antigens and bioactive substances that are produced by tumor cells following the abnormal expression of oncogenes, whereas under normal conditions, these markers are present in miniscule amounts or are absent. The high or low expression of tumor markers reflects changes in the expression of relevant genes during tumor progression 24. CEA is a conventional tumor biomarker that reflects tumor development, and CEA levels are significantly associated with lymphovascular invasion 25,26. The involvement of CEA in the adhesion of tumor cells to liver parenchyma is associated with liver metastasis 12. The combination of these two analyses can predict the prognosis of patients and is an important guide to the use of enteral nutrition and other methods to correct anemia in patients with advanced gastric cancer.

To our knowledge, this report describes a pilot study of HB-CEA as a novel scoring system. Herein, we demonstrated that the combination of hemoglobin and CEA levels is effective in predicting survival in patients with gastric cancer and that the predictive power of HB-CEA is superior to that of CEA or HB levels alone. However, there are a number of limitations of this study that need to be mentioned. Firstly, there is a need to increase the patient sample size and select patients from different geographic locations to reduce potential bias in retrospective study designs. Secondly, the postoperative treatment of patients can have an impact on the OS and could thus affect the diagnostic accuracy of the HB-CEA score. Thirdly, we were unable to fully assess the prognosis of patients with gastric cancer due to the inadequacy of data on disease-free survival. Therefore, the results of this study need to be validated with a larger sample size and analysis of more adequate clinicopathological and survival data from prospective studies.

## Conclusion

In this retrospective study, we demonstrated the high value of hemoglobin combined with CEA in the analysis of gastric cancer progression and patient prognosis. As a new scoring system, HB-CEA is of great significance for precise and individualized tumor treatment.

## Figures and Tables

**Table 1:** Prognostic Scores of HB, CEA and HB-CEA

Scoring System	Score
**Hemoglobin (g/L)**	
≥125.5	0
<125.5	1
**CEA (ng/mL)**	
<3.395	0
≥3.395	1
**Hemoglobin-CEA**	
HB≥125.5 and CEA<3.395	0
HB≥125.5 and CEA≥3.395	1
HB<125.5 and CEA<3.395	1
HB<125.5 and CEA≥3.395	2

Note: HB-CEA: the combination of HB and CEA. Abbreviations: HB: hemoglobin; CEA: carcinoembryonic antigen.

**Table 2:** The Clinicopathological Features of GC Patients

Characteristics	The training set							The validation set						
	Patients, n (%)							Patients, n (%)						
		Hemoglobin (g/L)			CEA (ng/mL)				Hemoglobin (g/L)			CEA (ng/mL)		
		≥125.5	<125.5	P value	<3.395	≥3.395	P value		≥125.5	<125.5	P value	<3.395	≥3.395	P value
**Sex**				<0.001*			0.010*				<0.001*			0.004*
Female	117(33.1%)	36	81		88	29		122(31.4%)	34	88		103	19	
Male	236(66.9%)	137	99		145	91		266(68.6%)	154	112		188	78	
**Age (years)**				0.428			0.108				0.002*			<0.001*
≤65	174(49.3%)	89	85		122	52		223(57.5%)	123	100		186	37	
>65	179 (50.7%)	84	95		111	68		165(42.5%)	65	100		105	60	
**Tumor location**				0.223			0.829				0.713			0.705
Upper third	80(22.7%)	44	36		52	28		71(18.3%)	33	38		52	19	
Middle and lower third	273(77.3%)	129	144		181	92		317(81.7%)	155	162		239	78	
**Differentiation**				0.041*			0.082				<0.001*			0.410
Moderate/Poor	330(93.5%)	157	173		214	116		343(88.4%)	152	191		255	88	
Well	23(6.5%)	16	7		19	4		45(11.6%)	36	9		36	9	
**Diameter of lesion (cm)**				0.003*			0.118				<0.001*			0.025*
<3	82(23.2%)	52	30		60	22		115(29.6%)	72	43		95	20	
≥3	271(76.8%)	121	150		173	98		273(70.4%)	116	157		196	77	
**Cancer embolism**				0.002*			0.583				0.484			0.401
None	242(68.6%)	132	110		162	80		345(88.9%)	165	180		261	84	
Yes	111(31.4%)	41	70		71	40		43(11.1%)	23	20		30	13	
**Nerve invasion**				0.697			0.784				0.494			0.275
None	227(64.3%)	113	114		151	76		333(85.8%)	159	174		253	80	
Yes	126(35.7%)	60	66		82	44		55(14.2%)	29	26		38	17	
**pTNM**				0.001*			0.006*				0.015*			0.003*
I-II	162(45.9%)	95	67		119	43		219(56.4%)	118	101		177	42	
III-IV	191(54.1%)	78	113		114	77		169(43.6%)	70	99		114	55	
**Depth of invasion**				<0.001*			0.002*				<0.001*			0.006*
T1-2	120(34.0%)	80	40		92	28		162(41.8%)	98	64		133	29	
T3-4	233(66.0%)	93	140		141	92		226(58.2%)	90	136		158	68	
**LN metastasis**				0.004*			0.011*				0.001*			0.002*
N0	123(34.8%)	73	50		92	31		168(43.3%)	97	71		139	29	
N1/N2/N3	230(65.2%)	100	130		141	89		220(56.7%)	91	129		152	68	
**Survival status**				<0.001*			<0.001*				<0.001*			<0.001*
Survival	221(62.6%)	130	91		163	58		241(62.1%)	136	105		200	41	
Death	132(37.4%)	43	89		70	62		147(37.9%)	52	95		91	56	

Note: HB-CEA: HB≥125.5 and CEA<3.395 represent 0; CEA≥3.395 or HB<125.5 represent 1; CEA≥3.395 and HB<125.5 represent 2. Abbreviations: GC: gastric cancer; CEA: carcinoembryonic antigen; HB: hemoglobin; pTNM: pathological TNM stage; LN metastasis: lymph node metastasis.

**Table 3:** The Clinicopathological Characteristics Stratified by the HB-CEA Score

Characteristics	The training set								The validation set			
	Before PSM				After PSM							
	HB-CEA	HB-CEA	HB-CEA	P value	HB-CEA	HB-CEA	HB-CEA	P value	HB-CEA	HB-CEA	HB-CEA	P value
	0	1	2		0	1	2		0	1	2	
**Sex**				0.231				0.388				0.037*
Female	33	58	26		20	40	26		31	75	16	
Male	84	114	38		44	88	38		103	136	27	
**Age (years)**				0.026*				0.165				<0.001*
≤65	59	93	22		30	62	22		98	113	12	
>65	58	79	42		34	66	42		36	98	31	
**Tumor location**				0.401				0.832				0.669
Upper third	31	34	15		18	33	15		24	37	10	
Middle and lower third	86	138	49		46	95	49		110	174	33	
**Differentiation**				0.014*				0.711				<0.001*
Moderate/Poor	103	165	62		62	126	62		107	193	43	
Well	14	7	2		2	2	2		27	18	0	
**Diameter of lesion (cm)**				0.005*				0.830				<0.001*
<3	36	40	6		8	13	6		57	53	5	
≥3	81	132	58		56	115	58		77	158	38	
**Cancer embolism**				0.037*				0.336				0.989
None	89	116	37		45	81	37		119	188	38	
Yes	28	56	27		19	47	27		15	23	5	
**Nerve invasion**				0.898				0.663				0.947
None	77	110	40		35	76	40		116	180	37	
Yes	40	62	24		29	52	24		18	31	6	
**pTNM**				<0.001*				0.380				<0.001*
I-II	71	72	19		25	38	19		94	107	18	
III-IV	46	100	45		39	90	45		40	104	25	
**Depth of invasion**				<0.001*				0.449				<0.001*
T1-2	60	52	8		13	19	8		82	67	13	
T3-4	57	120	56		51	109	56		52	144	30	
**LN metastasis**				0.001*				0.556				<0.001*
N0	55	55	13		18	29	13		79	78	11	
N1/N2/N3	62	117	51		46	99	51		55	133	32	
**Survival status**				<0.001*				<0.001*				<0.001*
Survival	95	103	23		47	77	23		108	120	13	
Death	22	69	41		17	51	41		26	91	30	

Note: HB-CEA: HB≥125.5 and CEA<3.395 represent 0; CEA≥3.395 or HB<125.5 represent 1; CEA≥3.395 and HB<125.5 represent 2. Abbreviations: CEA: carcinoembryonic antigen; HB: hemoglobin; pTNM: pathological TNM stage; LN metastasis: lymph node metastasis.

**Table 4:** Univariate and Multivariate Analyses of Clinicopathological Characteristics in GC Patients

Characteristics	The training set								The validation set			
	Before PSM				After PSM							
	Univariate Analysis		Multivariate Analysis		Univariate Analysis		Multivariate Analysis		Univariate Analysis		Multivariate Analysis	
	HR (95% CI)	P value	HR (95% CI)	P value	HR (95% CI)	P value	HR (95% CI)	P value	HR (95% CI)	P value	HR (95% CI)	P value
**Sex**		0.910				0.706				0.519		
Female	Ref				Ref				Ref			
Male	0.979 (0.683-1.404)				1.080 (0.724-1.611)				0.893 (0.632-1.260)			
**Age (years)**		0.082				0.107				0.030*		
≤65	Ref				Ref				Ref			
>65	1.357 (0.962-1.914)				1.373 (0.934-2.019)				1.430 (1.035-1.976)			
Tumor location		0.695				0.408				0.289		
Upper third	Ref				Ref				Ref			
Middle and lower third	1.084 (0.724-1.623)				1.200 (0.780-1.846)				0.805 (0.539-1.202)			
**Differentiation**		0.044*				0.271				0.005*		
Moderate/Poor	Ref				Ref				Ref			
Well	0.045 (0.002-0.916)				0.048 (0.001-10.681)				0.358 (0.176-0.731)			
**Diameter of lesion(cm)**		0.022*				0.895				<0.001*		
<3	Ref				Ref				Ref			
≥3	1.707 (1.080-2.698)				0.961 (0.527-1.751)				2.264 (1.491-3.437)			
**Cancer embolism**		<0.001*				0.001*				<0.001*		
None	Ref				Ref				Ref			
Yes	2.069 (1.468-2.917)				1.863 (1.279-2.713)				2.299 (1.492-3.542)			
**Nerve invasion**		0.007*				0.038*				<0.001*		0.043*
None	Ref				Ref				Ref		Ref	
Yes	1.607 (1.138-2.268)				1.490 (1.021-2.173)				2.241 (1.511-3.323)		1.525 (1.013-2.295)	
**pTNM**		<0.001*		0.014*		<0.001*		0.026*		<0.001*		0.004*
I-II	Ref		Ref		Ref		Ref		Ref		Ref	
III-IV	3.243 (2.193-4.797)		1.800 (1.125-2.881)		3.219 (1.938-5.348)		1.925 (1.082-3.423)		3.537 (2.511-4.982)		1.894 (1.225-2.928)	
**Depth of invasion**		<0.001*				0.040*				<0.001*		
T1-2	Ref				Ref				Ref			
T3-4	2.530 (1.636-3.912)				1.978 (1.032-3.792)				2.766 (1.905-4.018)			
**LN metastasis**		<0.001*		0.008*		<0.001*		0.004*		<0.001*		0.010*
N0	Ref		Ref		Ref		Ref		Ref		Ref	
N1/N2/N3	3.806 (2.385-6.075)		2.134 (1.217-3.741)		4.829 (2.437-9.569)		3.137 (1.444-6.813)		3.841 (2.591-5.694)		1.949 (1.175-3.233)	
**Hb levels (g/L)**		<0.001*				0.012*				<0.001*		
≥125.5	Ref				Ref				Ref			
<125.5	2.131 (1.481-3.067)				1.691 (1.124-2.545)				1.968 (1.402-2.761)			
**CEA levels (ng/mL)**		<0.001*				0.002*				<0.001*		
<3.395	Ref				Ref				Ref			
≥3.395	2.061 (1.463-2.903)				1.814 (1.245-2.643)				2.273 (1.628-3.173)			
**HB-CEA**												
0	Ref		Ref		Ref		Ref		Ref		Ref	
1	2.362 (1.462-3.817)	<0.001*	1.840 (1.134-2.985)	0.013*	1.615 (0.933-2.797)	0.087	1.411 (0.813-2.447)	0.221	2.585 (1.671-3.999)	<0.001*	2.049 (1.313-3.198)	0.002*
2	4.339 (2.583-7.289)	<0.001*	3.255 (1.927-5.500)	<0.001*	2.915 (1.655-5.134)	<0.001*	2.722 (1.544-4.800)	0.001*	5.427 (3.203-9.195)	<0.001*	4.007 (2.335-6.877)	<0.001*

Note: HB-CEA: HB≥125.5 and CEA<3.395 represent 0; CEA≥3.395 or HB<125.5 represent 1; CEA≥3.395 and HB<125.5 represent 2. Abbreviations: GC: gastric cancer; CEA: carcinoembryonic antigen; HB: hemoglobin; pTNM: pathological TNM stage; LN metastasis: lymph node metastasis; HR: hazard ratio; CI: confidence interval.
